# Entity-centric evaluation of large language model responses for medical question-answering tasks

**DOI:** 10.1371/journal.pdig.0001752

**Published:** 2026-10-06

**Authors:** Yi Liu, Vijaya B. Kolachalama

**Affiliations:** 1 Faculty of Computing & Data Sciences, Boston University, Boston, Massachusetts, United States of America; 2 Department of Medicine, Boston University Chobanian & Avedisian School of Medicine, Boston, Massachusetts, United States of America; 3 Department of Computer Science, Boston University, Boston, Massachusetts, United States of America; Liverpool John Moores University - City Campus: Liverpool John Moores University, UNITED KINGDOM OF GREAT BRITAIN AND NORTHERN IRELAND

## Abstract

Large language models (LLMs) are increasingly evaluated for clinical question-answering (QA) tasks, yet benchmark accuracy alone provides limited assurance of clinical reliability. Existing evaluation metrics often fail to capture whether model reasoning preserves patient-specific context and diagnostic intent, while many require external references or manual annotation, limiting scalability and real-world applicability. To address this gap, this study proposes EntQA, a reference-free, entity-centric metric that evaluates how well LLM-generated responses retain clinically relevant biomedical concepts from patient backgrounds and diagnostic questions. Across five medical QA benchmarks and seven Qwen 2.5 Instruct models (0.5B–72B parameters), EntQA showed consistently positive associations with model accuracy and scaling, outperforming conventional overlap- and embedding-based metrics that frequently exhibited weak or negative correlations. Group-level correlations with accuracy reached Spearman ρ =0.9286, while correlations with model scale reached ρ = 0.252. These findings suggest that EntQA provides a scalable and interpretable framework for assessing clinical fidelity and reasoning quality in healthcare LLMs without requiring gold standard references or external evidence.

## Author summary

Large language models (LLMs) are starting to appear in hospitals and clinics, where they help doctors answer questions and review patient information. However, these tools can sometimes miss important details from a patient’s history or the specific question being asked, which could lead to incomplete or misleading advice. Most current ways of checking these models only look at whether the final answer matches a reference. They often miss cases where the model ignored key clinical information from the original case. To help solve this problem, we created EntQA, a new way to evaluate how well LLM responses keep and use the important medical details from both the patient’s background and the question itself. EntQA works by identifying key medical concepts and checking which ones appear in the LLM’s answer. When we tested it across several well-known medical question-answering benchmarks, EntQA gave a clearer picture of how good the LLM responses really were compared with traditional scoring methods. It also makes it easier to see exactly which clinical details the model got right or missed. We believe tools like EntQA can help researchers and developers build safer, more reliable AI systems for healthcare.

## 1 Introduction

Large language models (LLMs) have demonstrated strong performance on a range of medical question answering (QA) tasks, including those that emulate licensing exams such as the USMLE. For instance, models like Med-PaLM [1] have achieved expert-level accuracy by exceeding passing thresholds on USMLE-style questions, showcasing their potential to assist in diagnostic reasoning and knowledge retrieval. However, evaluating medical LLMs remains challenging because physicians don’t judge medical advice solely by the correctness of the final diagnosis, but by whether the reasoning appropriately considers all clinically relevant patient information before arriving at a conclusion. In real-world clinical settings, overlooking seemingly minor details such as symptom duration, comorbidities or temporal progression can substantially alter diagnostic interpretation and potentially mislead clinical decision-making. Therefore, evaluation metrics for medical QA should assess whether model-generated responses remain grounded in the clinically relevant evidence provided in the patient case, rather than relying solely on the factual correctness of isolated statements or superficial textual similarity. Existing evaluation approaches often rely on coarse metrics such as accuracy or BLEU scores [2], which offer limited insight into whether model-generated responses faithfully adhere to patient-specific details. Consequently, these metrics may fail to identify cases where an LLM arrives at a seemingly correct conclusion while overlooking or fabricating critical clinical details from the patient case. This failure may allow clinically incomplete or insufficiently grounded reasoning to still receive favorable evaluation scores, potentially contributing to diagnostic errors and limiting the trustworthiness of such systems in high-stakes medical settings.

Prior work on evaluating LLMs in medical question answering initially focused on multiple-choice benchmarks such as MedQA [3] and USMLE-style tasks [4], where relying primarily on accuracy. Subsequent efforts expanded beyond exam-style setting to open-ended clinical tasks involving summarization, information extraction and diagnosis [5,6], as well as multilingual evaluation [7,8]. To assess those open-ended generation tasks, surface-level lexical metrics are adopted, such as BLEU [2], ROUGE [9] and embedding-based metrics (e.g., BERTScore [10]). However, these approaches depend on expert-written reference answers, which are costly and difficult to obtain in clinical settings. Moreover, recent studies have questioned the reliability of these automatic metrics (e.g., ROUGE, BERTScore) since they disagree with clinician preferences and expert quality assessments in medical summarization tasks [11].

As a result, recent work shifted toward rubric-based evaluation frameworks that assess whether model outputs satisfy clinically meaningful criteria. Several studies introduced detailed evaluation rubrics covering dimensions such as factual accuracy, completeness, clarity, contextual awareness, instruction adherence, clinical reasoning, and patient safety [12,13]. These frameworks typically rely on clinician experts or advanced LLM judges to score responses against predefined standards. For example, prior work employed physician annotations to evaluate the quality, credibility, and safety of medical LLM responses [14,15], while other studies proposed structured human evaluation protocols for assessing hallucination, factual consistency, diagnostic reasoning, and potential clinical harm [13,16].

Another line of work targets hallucination and reasoning validity by decomposing outputs into atomic facts or reasoning steps and verifying them against external knowledge bases [17–22]. These approaches first extract fine-grained factual claims either through manual annotation [17,22] or automated LLM-based decomposition [18,19], and then assess whether each claim is supported by external evidence. Verification is typically performed by comparing claims against source documents, retrieved web evidence, or structured medical knowledge graphs [19–21]. More recently, reasoning-oriented benchmarks have extended evaluation beyond factual correctness to assess the quality of intermediate clinical reasoning steps themselves, including their completeness, efficiency, and consistency with established medical knowledge [19].

Despite these advances, most methods remain dependent on external references, retrieval systems, or powerful proprietary LLMs, limiting scalability and interpretability. Importantly, they provide little insight into whether an LLM’s reasoning faithfully reflects patient-specific context and the diagnostic intent of the query, which are core requirements for safe clinical decision support. This gap motivates the need for the development of reference-free, entity-centric metrics that operate solely on the input context and generated response.

Here, this study introduced EntQA, an entity-centric metric tailored for medical QA tasks that involve patient background information and a diagnostic inquiry. EntQA operates solely on the existing task context, including the question, background, and LLM-generated responses, using biomedical named entity recognition to extract clinically relevant entities (e.g., symptoms, diagnoses, treatments) and query’s main intent. It then computes recall-style coverage from two perspectives: (1) $EntQA_{background}$ or $EntQA_{b}$ quantifies how well the response incorporates relevant contextual information from the patient’s clinical history, and (2) $EntQA_{question}$or $EntQA_{q}$ measures how effectively it addresses the diagnostic focus of the question. These recall scores provide interpretable insights into whether the LLM’s reasoning is adequately grounded in the patient’s context and the clinical inquiry, as the pipeline further alerts which key clinical entities are missing from the response, ensuring that responses remain trustworthy and free from topical drift. To foster proper evaluation, a dataset of LLM-generated responses for USMLE-style questions was curated. A meta-evaluation of EntQA against baseline metrics was conducted using this dataset and additional benchmarks.

## 2 Methods

EntQA, an entity-centric framework tailored for medical benchmark QA tasks, is proposed to quantify how effectively large language models (LLMs) interpret and incorporate clinically relevant information from input to their generated summaries. Unlike generic n-gram overlap metrics, EntQA explicitly measures the proportion of key medical entities, derived from either patient background or diagnostic question, that are preserved in the model’s response. This allows comparison of different LLM-generated summaries with respect to medical coverage and clinical reasoning fidelity.

As illustrated in Fig 1, the EntQA pipeline consists of three stages: (1) entity extraction and refinement, (2) entity semantic matching, and (3) coverage score computation. Importantly, because medical benchmark inputs are long and contain both patient background (B) and a real diagnostic question (Q), EntQA is designed from two complementary perspectives to evaluate LLM’s response (A): $EntQA_{background}$ using background as input and $EntQA_{question}$ using the question as input.

**Fig 1:**
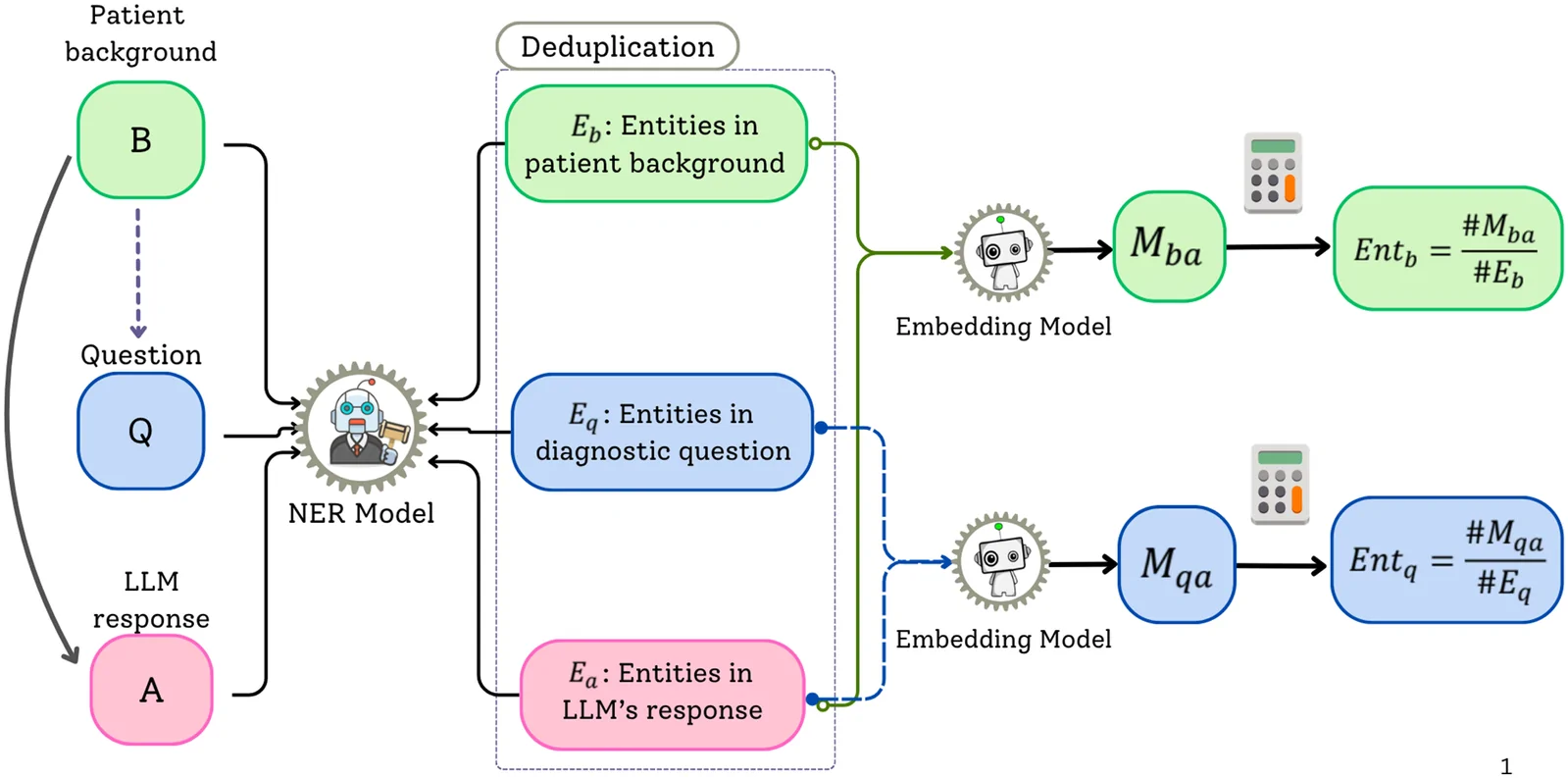
Schematic of the entity-based evaluation pipeline for LLM-generated responses. Named entity recognition (NER) extracts entities from the patient background ($E_{b}$), diagnostic question ($E_{q}$), and LLM response ($E_{a}$). Entities from all three sources undergo de-duplication before embedding. An embedding model computes semantic matches, yielding coverage metrics: the fraction of matched background entities in the response ($\frac{\#M_{ba}}{\#E_{b}}$) and matched question entities in the response ($\frac{\#M_{qa}}{\#E_{q}}$).

1 Structured entity extraction. The benchmark input was first separated into two components: the patient background (B) and the diagnostic question (Q). Clinically relevant entities were then extracted from the patient background, diagnostic question, and LLM-generated answer (A) using the BioEpidemiology-NER (BioEN) model proposed by Raza et al. [23]. BioEN is a Transformer-based biomedical NER framework built on DistilBERT for sequence labeling of clinical entities. The model was trained on multiple annotated biomedical and epidemiological corpora, primarily the MACROBBAT 2020 dataset, which contains 200 manually annotated PubMed clinical case reports spanning diverse entity categories such as diseases, symptoms, diagnostic procedures, medications, demographic information, temporal expressions, and epidemiological concepts. Prior work reported macro- and micro-average F1 scores above 90% across several biomedical NER benchmarks, indicating strong generalization capability for clinical text extraction tasks. Because the benchmark used in this study consists of patient histories, diagnostic narratives, and LLM-generated clinical reasoning outputs, BioEN is well suited for extracting clinically relevant entities in this setting. This step yields three sets of entities:
  - *E_b_*: entities extracted from the patient background,
  - *E_q_*: entities extracted from the diagnostic question,
  - *E_a_*: entities extracted from the LLM-generated answer.
Noise and redundancy in the raw extracted entities were further reduced by removing entities whose surface forms are equivalent up to punctuation or minor orthographic variation while retaining the longest mention; and by filtering out vague, repetitive, or generic entities introduced by multiple-choice question stem.
2 Entity semantic matching. Next, the extent to which input entities are faithfully represented in the LLM summary was assessed. A state-of-the-art embedding model [24] was employed to compute semantic similarity between entity pairs. Two types of matching were performed:
  - *M_ba_*: matches between entities in patient background *E_b_* and answer entities *E_a_*,
  - *M_qa_*: matches between entities in diagnostic question *E_q_* and answer entities *E_a_*,
An entity pair is considered matched if (i) their embedding similarity exceeds a threshold (e.g., cosine similarity *>* 0*.*7), and (ii) they share sufficient lexical overlap (e.g., token-level overlap *>* 30%). The lexical overlap criterion is introduced to mitigate false-positive matches that may arise when semantic similarity is assessed solely through embeddings. For example, pairs such as “39.1°C” and “90°C” or “965 ng/ml” and “83 mg/ml” pass the embedding-similarity criterion despite representing different measurements, while conceptually distinct entities such as “pelvic examination” and “pelvic inflammatory disease” still share a lexical overlap of 25%. A minimum token-level overlap of 30% therefore acts as a conservative filter, limiting spurious alignments and favoring matches that preserve the original entity mentioned rather than semantically related but non-equivalent entities. The overlap threshold can be adjusted according to dataset characteristics and the desired balance between matching precision and recall.
3 Coverage score computation. Finally, EntQA was formulated as a recall-style coverage measure
  - Background perspective:

$$\begin{array}{c} {EntQA}_{background}=\frac{\left|M_{ba}\right|}{\left|E_{b}\right|} \end{array}$$ (1)

- i.e., the proportion of entities from the patient background captured and represented in the LLM summary.
- Question perspective:

$$\begin{array}{c} {EntQA}_{question}=\frac{\left|M_{qa}\right|}{\left|E_{q}\right|} \end{array}$$ (2)

- i.e., the proportion of diagnostic question entities captured and represented in the LLM summary.

This score ranges from 0 to 1, where higher values indicate a greater degree of information interpretation and utilization. Importantly, the metric does not reward hallucinations or irrelevant details; it only quantifies how well the model retains and reflects essential source concepts.

By reporting both perspectives, EntQA not only assesses whether an LLM-generated summary adequately incorporates *contextual patient information* and addresses the *explicit diagnostic question*, but also enables users to explicitly trace which clinically important entities are preserved or omitted. This traceability provides additional information on the reliability of the summary, making EntQA a more clinically grounded and interpretable metric for benchmarking LLMs in medical QA tasks.

### 2.1 Experimental settings

To evaluate the reliability of LLMs as AI-assisted diagnostic tools, seven models from Qwen 2.5 Instruct family, which covers a wide range of parameter sizes from 0.5B to 72B, were employed. By deliberately covering a wide spectrum of parameter scales, the resulting model responses exhibited a gradient of reasoning abilities and answer qualities, ranging from shallow, error-prone rationales produced by small models to more coherent, clinically informed explanations from large models. This spectrum of outputs is critical for this study: it allows one to rigorously test whether the proposed metrics can reliably distinguish between high- and low-quality summaries by assessing how comprehensively patient-related entities are captured and reflected in the output as model capacity increases.

LLM-generated responses are collected over five medical QA benchmarks that are widely recognized in medical AI research: USMLE Step 1, USMLE Step 2, USMLE Step 3 [4], MedQA [3] and MedExpQA [7]. Each benchmark includes clinical case descriptions, multiple choice options, and the correct answers. Collectively, they form a clinically validated spectrum of test cases, covering preclinical sciences, case-based diagnostic reasoning, and advanced decision-making in patient care. The USMLE is a three-step licensing examination for medical practice in the United States. Step 1 tests core biomedical sciences such as physiology, pharmacology, and pathology (94 cases). Step 2 assesses clinical reasoning and patient management under supervision (109 cases). Step 3 evaluates advanced decision-making for independent practice, including diagnosis, prognosis, and treatment (122 cases). Compared with Step 1 and Step 2, Step 3 generally involves more complex clinical reasoning and requires integrating diagnostic findings, prognosis assessment, and treatment planning across longitudinal patient-care scenarios. Together, these steps form a progressive testbed for medical competence. MedQA is a curated benchmark collected after the USMLE board exams. The English subset used in this study contains 1,273 multiple-choice questions, aiming to assess a physician’s ability to apply medical knowledge, concepts and apply fundamental patient-centered skills. MedExpQA is derived from the Spanish national residency medical exam and contains 125 test cases with five answer choices and detailed explanations.

To ensure consistency of the generated output format across benchmarks, the prompt is standardized to elicit structured reasoning. Each question (patient information, question, and options) was presented to the model, followed by explicit instructions:

{background}{option}

Select the best answer and respond with:

“The correct answer is [OPTION LETTER].”

Then, write “Explanation:” and provide a detailed explanation, clearly stating why each option is correct or incorrect.

This prompt ensures that responses provide both a definitive answer and a comprehensive explanation, allowing a downstream evaluation of the coverage, informativeness, and diagnostic validity of the entity. For all LLMs, response generation was performed under a deterministic decoding configuration with temperature = 0, top_p = 1, and maximum output length set to 1024 tokens. These parameters minimize randomness in outputs and guarantee comparability across models and benchmarks.

To systematically evaluate whether the proposed metric can reliably assess the relative quality of multiple LLM responses to the same medical question, a series of experiments was conducted from two perspectives: (i) alignment with answer accuracy and (ii) alignment with model size as a proxy for LLM’s diagnostic reasoning ability. Rather than focusing solely on correlation strength, these analyses were used to investigate whether the proposed metric identifies summaries with greater clinical value, i.e., summaries that reflect patient information more comprehensively and are more aligned with accurate diagnostic reasoning. Furthermore, EntQA metrics were benchmarked against widely used n-gram overlap metrics (BLEU [2], ROUGE [9], METEOR [25]) to assess whether an entity-centric coverage approach better captures both the comprehensive interpretation of contextual information and the extent to which the model’s reasoning process incorporates the content of the question.

To assess whether the proposed metrics are aligned with model correctness trends, two complementary analyses were conducted: (1) Group-level correlation. For each benchmark, responses generated by the Qwen-2.5 Instruct models of increasing sizes were analyzed. For every model, the overall diagnostic accuracy and the average values of all evaluation metrics were computed, including the proposed entity-centric metrics for background coverage ($EntQA_{background}$ or $EntQA_{b}$) and question coverage ($EntQA_{question}$ or $EntQA_{q}$), as well as baseline metrics (BLEU, ROUGE, METEOR, and BERTScore [10]). The monotonic association between model accuracy and average metric scores was examined using Spearman and Kendall correlation coefficients. This analysis evaluates whether models with higher diagnostic accuracy also tend to produce responses receiving consistently higher metric scores, thereby reflecting alignment between metric evaluations and overall model correctness trends.

(2) Case-level correlation. To further evaluate the discriminative ability of the metrics at the individual response level, responses generated by models of different sizes were aggregated within each benchmark. For each response, the binary diagnostic correctness label (correct vs. incorrect) and the corresponding metric scores were collected, and the association between correctness labels and metric values was quantified using point-biserial correlation. This analysis assesses whether correct responses systematically receive higher metric scores than incorrect ones, thereby evaluating the ability of the metrics to distinguish correct predictions from incorrect predictions.

Beyond correctness alignment, the extent to which the metrics reflect response quality improvements as model capability increases was further investigated. Since model scale is commonly associated with stronger reasoning and generation ability, model size was used as a proxy for capability within the Qwen2.5 Instruct family. Specifically, for each benchmark, the average metric scores were computed across all responses generated by each model. Spearman correlation was then calculated between model size and model-level average metric scores. This analysis examines whether the metrics consistently assign higher scores to responses generated by larger and more capable models, thereby evaluating whether metric behavior remains aligned with expected improvements in reasoning quality under model scaling.

### 2.2 Computational hardware and software

All responses from the seven Qwen2.5 model variants across all medical benchmarks were generated locally on one or two NVIDIA L40 GPUs, depending on the model-specific computational requirements. Model inference was performed using openai (version 1.76.0) and vllm (version 0.8.4). Metric computation and statistical analysis were conducted in Python using pandas (version 2.2.2), numpy (version 1.26.4), evaluate (version 0.4.3), scipy (version 1.15.1). All figures were prepared using Canva.

## 3 Results

A qualitative case study from MedExpQA (Fig 2) is presented to illustrate the practical utility of EntQA in identifying clinically relevant overlaps and gaps in LLM-generated responses. The scenario describes a 47-year-old man with myopia magna who underwent cataract surgery 2 years ago and presented to the emergency room with profound and painless loss of vision in the right eye. The diagnostic question pertains to the most probable diagnosis, with options: (A) Post-surgical endophthalmitis, (B) Retinal detachment, (C) Age-related macular degeneration, wet form, (D) Posterior vitreous detachment. The model’s response suggests the correct answer “retinal detachment”, and the entity matching reveals the response retain most of the background information like “high myopia”, “cataract surgery”, “2 years”, “profound and painless loss”, “loss of vision”, while only omitting “emergency room”. In addition, the response covered one entity (“this symptomatology” as “this condition”). This yields coverage metrics of $EntQA_{b}=\text{0}.\text{8}\text{7}\text{5}$ (fraction of matched background entities in the answer) and $EntQA_{q}=\text{0}.\text{5}$ (fraction of matched question entities in the answer). By contrast, several lower EntQA responses for this question, whose scores are approximately 0.5-0.62), produced incorrect predictions, frequently selecting post-surgical endophthalmitis (option A) instead of option B. Some cases ignored “2 years” timeline, implicitly assuming the surgery was recent, thus incorrectly favoring a postoperative diagnosis. Others failed to reason about “high myopia”, a major risk factor for retinal detachment and a crucial clue supporting option B. These examples suggest that higher EntQA scores are generally associated with a more comprehensive use of clinically relevant contextual information and a higher probability of correct diagnostic reasoning.

**Fig 2:**
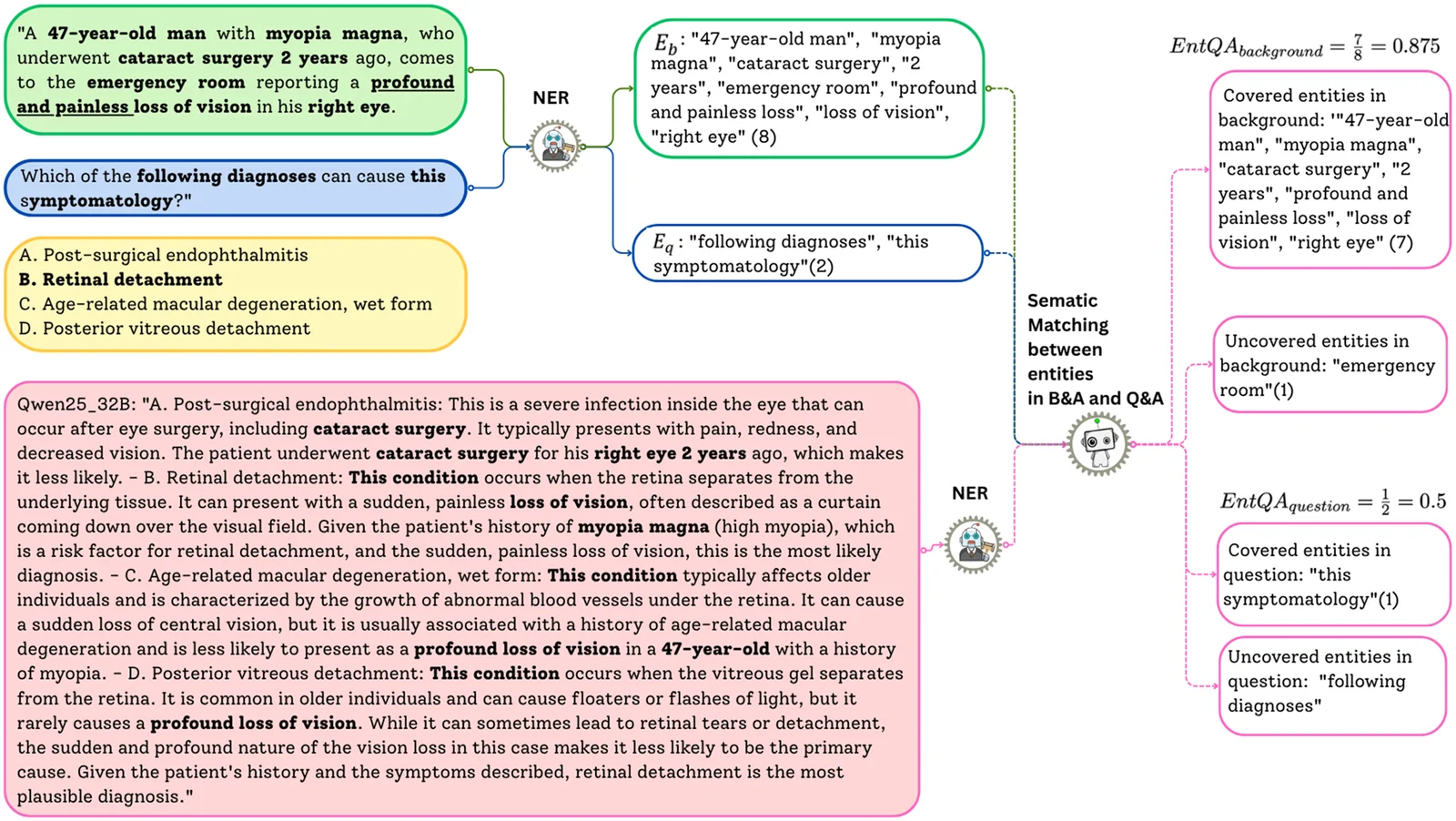
Case study from MedExpQA. This case demonstrates the entity-centric coverage metric’s ability to identify clinically relevant overlaps and gaps between the patient background, diagnostic question, and model-generated summary using biomedical named entity recognition (NER) and embedding-based entity matching.

### 3.1 Metric alignment with accuracy

At the group level, the proposed entity-centric metrics exhibit generally positive and often statistically significant alignment with model correctness across all five medical QA benchmarks (Table 1). Specifically, $EntQA_{b}$ shows consistently strong and significant positive correlations with accuracy: on MedQA, it achieves Spearman = 0.8571 (p<0.05) and Kendall = 0.7143 (p<0.05); on MedExpQA, it reaches the highest values among all metrics (Spearman = 0.9286, Kendall = 0.8095, p<0.01); and it remains strongly positive on USMLE Step 1 (0.8571/ 0.7143) and Step 3 (0.9286/ 0.8095). On USMLE Step 2, although the correlations do not reach statistical significance, $EntQA_{b}$ still exhibits a positive association with model accuracy (0.7143/ 0.619), whereas most baseline metrics remain negative. The $EntQA_{q}$ metric also maintains positive trends (e.g., 0.6429 on MedQA, 0.7143 on Step 3), suggesting that it effectively captures how well the generated answers align with the key entities in the question and is strengthened as the model’s final prediction accuracy improves. By contrast, all baseline metrics, i.e., BLEU, ROUGE, METEOR, and BERTScore, show predominantly negative correlation with accuracy, often at statistically significant levels (e.g., ROUGE-1 get -0.8929 correlation on MedQA; METEOR’s correlation is -0.9286 on USMLE STEP 1; BERTScore’s correlation is -0.9286 on STEP 1 and -0.8571 on STEP 3). These results highlight that conventional surface-matching metrics often misalign with accuracy, whereas the entity-centric metrics provide a more faithful reflection of model reliability.

**Table 1:** Correlation between model-level accuracy and average metric scores across medical benchmarks. This table presents Spearman and Kendall correlation coefficients between model-level accuracy and average scores for various metrics on datasets including MedQA, MedExpQA, USMLE Step 1, USMLE Step 2, and USMLE Step 3. Metrics evaluated include entity-based coverage (${EntQA}_{b}$ and ${EntQA}_{q}$), lexical overlap measures (BLEU-1, BLEU-4, ROUGE-1, ROUGE-2, ROUGE-L, METEOR), and semantic similarity (BERTScore-F1). Significance levels are indicated by * (p<0.05) and ** (p<0.01), highlighting the strength and direction of associations, with positive correlations for entity-centric metrics and often negative for traditional n-gram based metrics.

Metric	MedQA		MedExpQA		USMLE_Step_1		USMLE_Step_2		USMLE_Step_3	
	Spearman	Kendall	Spearman	Kendall	Spearman	Kendall	Spearman	Kendall	Spearman	Kendall
$EntQA_{b}$	0.8571*	0.7143*	0.9286**	0.8095*	0.8571*	0.7143*	0.7143	0.619	0.9286**	0.8095*
$EntQA_{q}$	0.6429	0.4286	0.5357	0.4286	0.6429	0.5238	0.2143	0.1429	0.7143	0.5238
BLEU-1	-0.8571*	-0.7143*	-0.7143	-0.619	-0.8214*	-0.7143*	-0.8571*	-0.7143*	-0.6429	-0.4286
BLEU-4	0.4643	0.3333	-0.25	-0.1429	-0.7857*	-0.619	-0.4286	-0.3333	-0.1786	-0.1429
ROUGE-1	-0.8929**	-0.8095*	-0.8571*	-0.7143*	-0.8214*	-0.7143*	-0.8929**	-0.8095*	-0.6786	-0.5238
ROUGE-2	-0.2857	-0.2381	-0.3929	-0.3333	-0.9286**	-0.8095*	-0.8929**	-0.7143*	-0.75	-0.5238
ROUGE-L	-0.8214*	-0.6190	-0.6429	-0.5238	-0.7857*	-0.619	-0.8571*	-0.7143*	-0.6429	-0.4286
METEOR	-0.8571*	-0.7143*	-0.6429	-0.5238	-0.9286**	-0.8095*	-1**	-1**	-0.75	-0.5238
BERTScore-F1	-0.8929**	-0.8095*	-0.8929**	-0.8095*	-0.9286**	-0.8095*	-0.8929**	-0.8095*	-0.8571*	-0.7143*

To further examine these findings beyond the aggregated model-group analysis, Table 2 reports point-biserial correlations between metric scores and per-case correctness labels. Overall, the two proposed EntQA metrics exhibit more consistently positive associations with accuracy across the five benchmarks, whereas the conventional baseline metrics often show weak, negative, or inconsistent correlations. Specifically, $EntQA_{b}$ yields positive correlations on all five datasets, reaching statistical significance on MedQA (r=0.0595, p<0.01), MedExpQA (r=0.0926, p<0.01), and USMLE Step 3 (r=0.090, p<0.01). Similarly, $EntQA_{q}$ remains positive across all benchmarks and achieves significance on both MedQA (r=0.0373, p<0.01) and USMLE Step 3 (r=0.0729, p<0.05). In contrast, several overlap-based baseline metrics, including ROUGE-1, ROUGE-2, ROUGE-L, and BERTScore-F1, show negative correlations on multiple benchmarks (e.g., ROUGE-1 on USMLE Step 1: r=−0.1105, p<0.01; BERTScore-F1 on USMLE Step 1: r=−0.1287, p<0.01). BLEU-4 is the only baseline metric exhibiting a positive and significant correlation on MedExpQA (r=0.0987, p<0.01), but this pattern is not consistently maintained across the remaining benchmarks. Although the observed point-biserial correlations are modest in magnitude, the proposed entity-centric metrics exhibit more stable and consistently positive associations with correctness across benchmarks than traditional overlap-based metrics. This pattern suggests that entity-centric metrics provide more reliable directional alignment with case-level correctness, despite their limited predictive strength at the individual-case level.

**Table 2:** Point-biserial correlation between per-example accuracy labels and metric scores across medical benchmarks. This table presents point-biserial correlation coefficients (r) and their corresponding p-values between per-example accuracy labels and scores for various metrics on datasets including MedQA, MedExpQA, USMLE Step 1, USMLE Step 2, and USMLE Step 3. Metrics evaluated include entity-based coverage ($EntQA_{b}$ and $EntQA_{q}$), lexical overlap measures (BLEU-1, BLEU-4, ROUGE-1, ROUGE-2, ROUGE-L, METEOR), and semantic similarity (BERTScore F1). Significance levels are indicated by * (p<0.05) and ** (p<0.01), showing generally positive correlations for entity-centric metrics and mixed or negative associations for traditional *n*-gram based metrics.

Metric	MedQA		MedExpQA		USMLE_Step_1		USMLE_Step_2		USMLE_Step_3	
	r	p	r	p	r	p	r	p	r	p
$EntQA_{b}$	0.0595^**^	0.0	0.0926^**^	0.0061	0.0129	0.7425	0.0394	0.2769	0.09^**^	0.0085
$EntQA_{q}$	0.0373^**^	0.0004	0.0222	0.5113	0.0055	0.889	-0.0236	0.5159	0.0729^*^	0.0332
BLEU-1	-0.0593^**^	0.0	-0.0068	0.8410	-0.0977^*^	0.0123	-0.1153^**^	0.0014	0.0071	0.8367
BLEU-4	0.0113	0.2866	0.0987^**^	0.0035	-0.0389	0.3196	-0.0327	0.3676	0.0081	0.8140
ROUGE-1	-0.0667^**^	0.0	-0.0050	0.8828	-0.1105^**^	0.0046	-0.1517^**^	0.0000	-0.0113	0.7408
ROUGE-2	-0.0028	0.7905	0.0532	0.1156	-0.0596	0.1274	-0.1092^**^	0.0025	-0.0011	0.9746
ROUGE-L	-0.0425^**^	0.0001	0.0206	0.5419	-0.1050^**^	0.0071	-0.1434^**^	0.0001	-0.0171	0.6183
METEOR	-0.0242^*^	0.0225	0.0273	0.4201	-0.0533	0.1724	-0.1373^**^	0.0001	-0.0005	0.9875
BERTScore F1	-0.0826^**^	0.0	-0.0435	0.1991	-0.1287^**^	0.0010	-0.1018^**^	0.0049	-0.0639	0.0622

### 3.2 Metrics under model scaling

The proposed entity-centric metrics exhibit consistent positive alignment with model scaling across all five medical QA benchmarks (Table 3), indicating that EntQA better captures improvements in the quality of responses generated by larger models, which are generally associated with stronger reasoning ability, than conventional overlap-based metrics. $EntQA_{b}$shows significant positive correlations with model size on every dataset, including 0.188 on MedQA, 0.181 on MedExpQA, 0.123 on Step 1, 0.171 on Step 2, and 0.252 on Step 3, demonstrating that background-level entity coverage systematically improves as models become larger and more capable. $EntQA_{q}$ also trends positive, reaching significance on MedQA (r=0.079, p<0.01), MedExpQA (r=0.081, p<0.05), Step 1 (r=0.086, p<0.05), and Step 3 (r=0.111, p<0.01), though with smaller effect sizes. In contrast, baseline metrics are largely misaligned with scaling: BLEU-1 (r=−0.192, p<0.01), ROUGE-1 (r=−0.216, p<0.01), ROUGE-L (r=−0.152, p<0.01), METEOR (r=−0.089, p<0.01), and BERTScore-F1 (r=−0.333, p<0.01) all exhibit strong negative correlations on MedQA, with similar negative patterns across other benchmarks (e.g., BERTScore-F1, r=−0.425, p<0.01 on Step 1). BLEU-4 shows only weak and inconsistent positives, i.e., 0.072 on MedQA and 0.091 on MedExpQA, while ROUGE-2 remains near zero throughout. These results confirm that entity-centric coverage metrics, especially $EntQA_{b}$, are the only measures that consistently track systematic quality improvements as model scale increases, whereas conventional overlap-based metrics fail to do so.

**Table 3:** Metric alignment with model scale: Spearman and Kendall correlation analysis across medical benchmarks. This table presents Spearman and Kendall correlation coefficients assessing the relationship between metric scores and model scale on datasets including MedQA, MedExpQA, USMLE Step 1, USMLE Step 2, and USMLE Step 3. Metrics evaluated include entity-based coverage ($EntQA_{b}$and $EntQA_{q}$), lexical overlap measures (BLEU-1, BLEU-4, ROUGE-1, ROUGE-2, ROUGE-L, METEOR), and semantic similarity (BERTScore-F1). Significance levels are indicated by * (p<0.05) and ** (p<0.01), revealing generally positive correlations for entity-centric metrics and negative associations for most traditional n-gram based metrics, suggesting stronger alignment of entity-based evaluations with increasing model scale.

Metric	MedQA		MedExpQA		USMLE_Step_1		USMLE_Step_2		USMLE_Step_3	
	Spearman	Kendall	Spearman	Kendall	Spearman	Kendall	Spearman	Kendall	Spearman	Kendall
$EntQA_{b}$	0.188^**^	0.137^**^	0.181^**^	0.132^**^	0.123^**^	0.089^**^	0.171^**^	0.127^**^	0.252^**^	0.184^*^
$EntQA_{q}$	0.079^**^	0.104^**^	0.081^*^	0.064^*^	0.086^*^	0.066*	0.018	0.015	0.111**	0.086**
BLEU-1	-0.192**	-0.138**	-0.106**	-0.076**	-0.331**	-0.241**	-0.219**	-0.158**	-0.177**	-0.128**
BLEU-4	0.072**	0.054**	0.091**	0.073**	-0.031	-0.027	-0.042	-0.033	0.042	0.026
ROUGE-1	-0.216**	-0.156*	-0.113**	-0.082**	-0.359**	-0.261**	-0.244**	-0.176**	-0.213**	-0.154**
ROUGE-2	-0.003	-0.003	-0.002	-0.003*	-0.152**	-0.111*	-0.137**	-0.1*	-0.024	-0.02
ROUGE-L	-0.152**	-0.109**	-0.088**	-0.064**	-0.306**	-0.224**	-0.207**	-0.149**	-0.117**	-0.084**
METEOR	-0.089**	-0.064**	-0.06	-0.043	-0.218**	-0.158**	-0.192**	-0.138**	-0.09**	-0.065**
BERTScore-F1	-0.333**	-0.241*	-0.231**	-0.166**	-0.425**	-0.312**	-0.309**	-0.223**	-0.303**	-0.221**

## 4 Discussion

EntQA, a novel entity-centric metric, was designed to evaluate the clinical alignment and informativeness of LLM-generated responses in medical question-answering (QA) tasks. By leveraging biomedical named entity recognition (NER), semantic embedding-based matching, and coverage score computation, EntQA quantifies how well key entities from patient backgrounds and diagnostic questions are preserved in model outputs, addressing gaps in traditional metrics like accuracy, BLEU, ROUGE, METEOR, and BERTScore. Experiments on five benchmarks using Qwen 2.5 Instruct models of varying scales shows that EntQA exhibits strong positive correlations with model accuracy at both groups (Spearman up to 0.9286) and per-case levels (point-biserial up to 0.0926), as well as with model scaling (Spearman up to 0.252). In contrast, baseline metrics often showed negative or inconsistent alignments, underscoring EntQA’s superiority in capturing clinically relevant response quality. EntQA is lightweight, domain-adapted via biomedical NER, and operates without external knowledge bases, making it especially suitable for long-form clinical narratives. It also complements trustworthiness checklists proposed for healthcare AI by providing explicit, traceable entity-level grounding that clinicians can directly inspect.

EntQA complements and advances existing evaluation frameworks in medical AI, such as those focused on multi-label classification metrics like precision, recall and AUC-ROC, which are prevalent in diagnostic tasks. While these metrics excel in discriminative models, they fall short for generative LLMs, where n-gram-based baselines like BLEU and ROUGE often exhibit negative correlations with accuracy due to their sensitivity to surface-level variations rather than clinical grounding. In contrast, EntQA’s entity-centric approach mitigates common pitfalls, such as overlooking contextual omissions or hallucinations, aligning more closely with trustworthiness metrics proposed for healthcare conversations that emphasize domainspecific fidelity and user-centric evaluation. Compared with recent rubric-based evaluation frameworks and hallucination-oriented metrics, EntQA does not rely on expert-written references, proprietary LLM judges, or external medical knowledge bases. Instead, it performs entity-level matching and coverage analysis directly on the patient background, diagnostic question, and generated response, providing an interpretable assessment of clinical grounding and patient-specific reasoning.

The implications of EntQA extend beyond mere benchmarking, offering a pathway to more trustworthy AI-assisted diagnostics in healthcare. By providing interpretable insights into entity omissions (low recall) and hallucinations (low precision), EntQA enables clinicians, researchers and developers to pinpoint deficiencies in LLM reasoning, such as overlooking patient-specific details like symptoms or medical history, which could otherwise lead to diagnostic errors. This metric’s focus on topical consistency and grounding in input context supports the development of LLMs that prioritize patient-centered explanations over generic responses, potentially enhancing their utility in real-world clinical workflows, such as decision support systems or educational tools. Furthermore, its extensions to fact-checking and model improvement through entity-conditioned question regeneration, could facilitate iterative fine-tuning of LLMs, promoting safer deployment in high-stakes environments.

EntQA has some limitations. First, its reliance on a biomedical NER model introduces potential biases or errors in entity extraction, particularly for rare or contextually ambiguous medical terms, which could affect coverage scores. The semantic matching thresholds (e.g., cosine similarity *>* 0*.*7 and lexical overlap *>* 30%) may require dataset-specific tuning to optimize performance. Importantly, EntQA is intentionally designed to evaluate entity-level grounding and information coverage, focusing on whether clinically relevant details from patient backgrounds and diagnostic questions are adequately reflected in model-generated responses. This design is motivated by a common failure mode in medical LLMs, where models may generate plausible or fluent answers while overlooking critical patient-specific information, potentially leading to biased or incomplete clinical conclusions. In this context, low EntQA scores can help identify responses that fail to sufficiently incorporate key clinical evidence from the input. However, because EntQA focuses primarily on information retention and topical grounding, it does not fully capture higher-level aspects of response quality, such as logical coherence, causal reasoning, or factual correctness of medical interpretations. Consequently, responses preserving most of the contextual entities or with high entity coverage may still contain flawed reasoning or incorrect interpretations. EntQA should therefore be viewed as a complementary evaluation metric rather than a standalone assessment framework, and is most effective when combined with additional evaluation approaches such as reasoning-focused metrics, factuality assessment, structured error analysis, or expert clinician review. Additionally, our evaluation was conducted on a specific family of models (i.e., Qwen 2.5) and English-centric benchmarks, limiting generalizability to multilingual settings, other LLM architectures, or non-QA clinical tasks such as summarization or dialogue. Future work could integrate EntQA into standardized checklists for AI studies, ensuring reproducibility through open source code and diverse datasets. Additionally, extending EntQA to multimodal tasks (e.g., incorporating imaging or lab data) or combining it with multi-dimensional frameworks, would enhance its applicability to real-world healthcare AI systems.

## 5 Conclusion

This study introduces EntQA, a scalable, reference-free, entity-centric metric that evaluates clinical fidelity in LLM-generated medical responses by measuring retention of patient-specific and diagnostic entities. Across five established benchmarks and seven Qwen 2.5 Instruct models spanning three orders of magnitude in scale, EntQA demonstrated consistently positive and often statistically significant associations with both answer accuracy and model scaling, outperforming conventional lexical and embedding-based metrics. By delivering interpretable entity-level insights into omissions and contextual grounding, EntQA provides a practical tool for developers, researchers, and clinicians to assess and iteratively improve the trustworthiness of medical LLMs. The framework advances the development of clinically reliable AI systems and underscores the value of entity-centric evaluation in high-stakes healthcare domains.

## Supporting information

S1 AppendixSensitivity analyses of entity matching thresholds in EntQA pipeline; and illustration of the EntQA pipeline.Fig A–E in S1 Appendix. Sensitivity analysis of entity matching thresholds in the EntQA pipeline. These figures assess the robustness of EntQA to variations in entity matching parameters. Across a wide range of embedding similarity and lexical overlap thresholds, EntQA generally maintains stable positive associations with model accuracy and model scaling trends across five biomedical question-answering benchmarks. Fig F in S1 Appendix. Illustration of the EntQA workflow using a MedQA case study.(DOCX)
