# Supplementary material for: Entity-centric evaluation of large language model responses for medical question-answering tasks
Source: PLOS Digit Health. 2026 Oct 6;5(10):e0001752. doi: 10.1371/journal.pdig.0001752 (PMC13641718; doi:10.1371/journal.pdig.0001752)
Supplement: Supplementary file 1 [file pdig.0001752.s003.pdf]

Reviewers' comments:

Reviewer #1:

Comment R1C1: In the manuscript, you state that for the system to perform semantic matching, "the cosine similarity must be greater than 0.7 and the word overlap must be over 30%." However, I did not see an analysis showing how changing these values affects EntQA's performance. I noted that you mentioned these thresholds might require dataset-specific tuning to optimize performance, but I am left wondering whether these parameters manipulate the results and, in datasets where there are no definitive truths, how these adjustments should be made.

Response R1C1: We appreciate the reviewer's concerns. We would first like to clarify that the manuscript does not state that the system universally requires a cosine similarity threshold of 0.7 and lexical overlap threshold of 30%. Rather, the manuscript states:

> "An entity pair is considered matched if (i) their embedding similarity exceeds a threshold (e.g., cosine similarity > 0.7), and (ii) they share sufficient lexical overlap (e.g., token-level overlap >30%)."

Here, "e.g." was intended to indicate example operating points rather than fixed universally optimal settings. We also explicitly noted that these thresholds may require dataset-specific tuning.

We agree that threshold selection can influence the absolute values of the reported correlations. To address this concern, we conducted an additional sensitivity analysis across a range of embedding similarity and lexical overlap thresholds (new Appendix Figure S1-S5). Although the exact correlation magnitudes vary across parameter settings, the principal conclusions remain unchanged. Across threshold combinations, EntQA metrics consistently demonstrate stronger and more positive associations with model size and answer correctness than the baseline metrics, whose correlations remain predominantly negative. These results indicate that the conclusions are robust and not dependent on a narrowly optimized parameter choice.

Regarding the lexical overlap threshold specifically, this constraint was introduced to reduce clinically implausible semantic matches that can arise when relying solely on embedding similarity. For example, embedding similarity alone may incorrectly match pairs such as:

- "pelvic examination" ↔ "pelvic inflammatory disease"(lexical overlap = 0.25),
- "39.1°C" ↔ "90°C"(lexical overlap = 0),
- "Poor score" ↔ "behavioral issues" (lexical overlap = 0),
- or "965 ng/ml" ↔ "83 mg/ml", (lexical overlap = 0)

despite these pairs representing distinct or contradictory clinical concepts. Regarding the selection of the lexical overlap threshold, we have added further clarification in the Methods section (at the end of '2. Entity semantic matching.' part).

An entity pair is considered matched if (i) their embedding similarity exceeds a threshold (e.g., cosine similarity > 0.7), and (ii) they share sufficient lexical overlap (e.g., token-level overlap >30%). The lexical overlap criterion is introduced to mitigate false-positive matches that may

arise when semantic similarity is assessed solely through embeddings. For example, pairs such as "39.1°C" and "90°C" or "965 ng/ml" and "83 mg/ml" pass the embedding-similarity criterion despite representing different measurements, while conceptually distinct entities such as "pelvic examination" and "pelvic inflammatory disease" still share a lexical overlap of 25%. A minimum token-level overlap of 30% therefore acts as a conservative filter, limiting spurious alignments and favoring matches that preserve the original entity mentioned rather than semantically related but non-equivalent entities. The overlap threshold can be adjusted according to dataset characteristics and the desired balance between matching precision and recall.

Accordingly, the thresholds used in the main experiments were selected as a clinically interpretable and conservative operating point that balances semantic flexibility with matching precision, rather than as parameters optimized to maximize the reported correlations.

Comment R1C2: In the article, the r value for EntQAb in the USMLE Step 3 is reported as 0.0490, but Table 2 lists it as 0.09. Which one is correct?

Response R1C2: The value reported in Table 2 ($r = 0.09$, $p < 0.01$) is correct. The value in the main text ($r = 0.0490$) was incorrectly reported. We have corrected this value in the main text, and also carefully checked all numerical values to ensure consistency throughout the manuscript.

Comment R1C3: Do you have any explanation for why BLEU-4 shows a higher correlation in the case-level accuracy test?

Response R1C3: The relatively higher correlation of BLEU-4 on MedExpQA (corr = 0.0987**) appears to be dataset-specific rather than consistently robust. In Table 2, the reported correlations are computed on the pooled responses from seven Qwen 2.5 model variants. On MedExpQA, when the seven Qwen 2.5 models are examined separately, the mean BLEU-4 score of correct responses exceeds that of incorrect responses for all models, yielding uniformly positive score differences. This consistent pattern is subsequently reflected in the pooled analysis, resulting in a relatively higher aggregated correlation.

In contrast, BLEU-4 does not show the same behavior on other benchmarks. For example, on MedQA (corr = 0.0113), only four of the seven Qwen 2.5 models exhibit higher BLEU-4 scores for correct responses than for incorrect ones. Similarly, on USMLE Step 3 (corr = 0.0081), although six models show positive differences, the magnitudes are extremely small and close to zero. As a result, BLEU-4 cannot consistently distinguish correct from incorrect responses on these benchmarks, leading to weak and statistically insignificant case-level correlations.

These findings suggest that the stronger correlation observed on MedExpQA is largely an incidental result of benchmark-specific response patterns rather than a consistently reliable property of BLEU-4. In contrast, the proposed entity-centric metrics demonstrate more stable associations with response correctness across datasets and model scales.

[MedExpQA]

model	correct_response	incorrect_response	metric_difference
0.5B-Qwen2.5	0.0033	0.0019	0.0014
1.5B-Qwen2.5	0.0030	0.0017	0.0014
3B-Qwen2.5	0.0042	0.0023	0.0019
7B-Qwen2.5	0.0024	0.0023	0.0019
14B-Qwen2.5	0.0025	0.0017	0.0008
32B-Qwen2.5	0.0035	0.0016	0.0019
72B-Qwen2.5	0.0024	0.0007	0.0017

[MedQA]

model	correct_response	incorrect_response	metric_difference
0.5B-Qwen2.5	0.0044	0.0047	-0.0003
1.5B-Qwen2.5	0.0048	0.0046	0.0002
3B-Qwen2.5	0.0071	0.0060	0.0011
7B-Qwen2.5	0.0053	0.0053	0.0000
14B-Qwen2.5	0.0049	0.0045	0.0004
32B-Qwen2.5	0.0052	0.0056	-0.0004
72B-Qwen2.5	0.0049	0.0052	-0.0003

[USMLE_STEP_3]

model	correct_response	incorrect_response	metric_difference
0.5B-Qwen2.5	0.0058	0.0080	-0.0022
1.5B-Qwen2.5	0.0087	0.0074	0.0013
3B-Qwen2.5	0.0112	0.0097	0.0015
7B-Qwen2.5	0.0076	0.0072	0.0004
14B-Qwen2.5	0.0082	0.0080	0.0002
32B-Qwen2.5	0.0081	0.0078	0.0002

72B-Qwen2.5	0.0070	0.0067	0.0003

Comment R1C4: Although you have noted that the results are weak and inconsistent, even the highest EntQA correlation in your point-biserial correlation table remains below 0.1. Yes, the p-value appears statistically significant, but I believe this may be related to the sample size. Therefore, I think it would be more accurate to evaluate the results from this perspective.

Response R1C4: We agree that the observed point-biserial correlations are modest in magnitude and should be interpreted cautiously, regardless of statistical significance. While some statistically significant results may benefit from the aggregated sample size obtained by pooling responses from multiple models, our intention is not to claim strong predictive power at the case level.

Rather, the purpose of this analysis is to examine whether an evaluation metric exhibits a consistent directional association with correctness across benchmarks and model scales. From this perspective, the key observation is not the absolute magnitude of the correlations, but their consistency in direction. The proposed entity-centric metrics show positive associations with correctness across nearly all benchmarks, whereas several baseline metrics exhibit near-zero, negative, or inconsistent correlations under the same evaluation setting.

We further note that similar correlation magnitudes are observed across different entity-matching thresholds (Figures S1–S5), suggesting that these modest effect sizes are not driven by a particular threshold choice. Instead, they likely reflect the inherent difficulty of per-example evaluation in medical QA, where correctness is influenced by multiple factors beyond lexical or entity overlap.

To address this concern, we have revised the manuscript to explicitly acknowledge the modest effect sizes and clarify the interpretation of Table 2 accordingly.

> *In contrast, several overlap-based baseline metrics, including ROUGE-1, ROUGE-2, ROUGE-L, and BERTScore-F1, show negative correlations on multiple benchmarks (e.g., ROUGE-1 on USMLE Step 1: $r=-0.1105$, $p<0.01$; BERTScore-F1 on USMLE Step 1: $r=-0.1287$, $p<0.01$). BLEU-4 is the only baseline metric exhibiting a positive and significant correlation on MedExpQA ($r=0.0987$, $p<0.01$), but this pattern is not consistently maintained across the remaining benchmarks. Although the observed point-biserial correlations are modest in magnitude, the proposed entity-centric metrics exhibit more stable and consistently positive associations with correctness across benchmarks than traditional overlap-based metrics. This pattern suggests that entity-centric metrics provide more reliable directional alignment with case-level correctness, despite their limited predictive strength at the individual-case level.*

Comment R1C5: Additionally, an assessment is needed regarding the lack of significant correlation in USMLE Step 1 and 2 in Table 2. I couldn't make sense of the difference between them and Step 3.

Response R1C5: We agree that the weaker and non-significant correlations observed on USMLE Step 1 and Step 2 warrant further discussion. One possible explanation relates to the differing clinical focus of the three USMLE stages. Step 1 primarily evaluates foundational biomedical knowledge, such as physiology, pathology, and pharmacology, where correctness is often determined by identifying a specific factual concept or mechanism. Although Step 2 assesses clinical reasoning and patient management, many questions focus on selecting the most appropriate diagnostic or management decision based on a limited number of key clinical cues. In these settings, a response may be correct without requiring extensive coverage of clinical entities.

In contrast, Step 3 emphasizes advanced clinical decision-making for independent practice, including diagnosis, prognosis, and treatment planning in more complex patient-care scenarios. Correct responses therefore often require integrating multiple clinically relevant entities and relationships within a coherent reasoning process. Under such conditions, broader clinical entity coverage may be more closely associated with response correctness, which could explain the stronger positive correlations observed between EntQA and performance on Step 3.

To clarify this point, we have revised the Experimental Setting section by adding a description of the differing objectives and complexity levels of USMLE Step 1, Step 2, and Step 3 after the five benchmarks information.

> *Compared with Step 1 and Step 2, Step 3 generally involves more complex clinical reasoning and requires integrating diagnostic findings, prognosis assessment, and treatment planning across longitudinal patient-care scenarios.*

Comment R1C6: Additionally, in the article, you state that "EntQAb still maintains a positive trend (0.7143 / 0.619)," but it is clear from Table 1 that no statistical significance is found.
Response R1C6: We agree that the original wording was not sufficiently precise regarding the distinction between positive trends and statistically significant findings, particularly for USMLE Step 2 where the correlations are positive but do not reach statistical significance. In the revised manuscript, we have toned down the corresponding statements to more accurately reflect the results.

> *At the group level, the proposed entity-centric metrics exhibit generally positive and often statistically significant alignment with model correctness across all five medical QA benchmarks (Table 1). Specifically, EntQA_b shows consistently strong and significant positive correlations with accuracy: on MedQA, it achieves Spearman = 0.8571 ($p<0.05$) and Kendall = 0.7143 ($p<0.05$); on MedExpQA, it reaches the highest values among all metrics (Spearman = 0.9286, Kendall = 0.8095, $p<0.01$); and it remains strongly positive on USMLE Step 1 (0.8571 / 0.7143) and Step 3 (0.9286 / 0.8095). On USMLE Step 2, although the correlations do not reach statistical significance, EntQA_b still exhibits a positive association with model accuracy (0.7143 / 0.619), whereas most baseline metrics remain negative.*

Comment R1C7: In the Introduction section, on lines 41–42, you have used the phrase "where it consistently showed higher correlation with model accuracy and reasoning ability." It would be more appropriate to include this in the Results and/or Discussion section.

Response R1C7: We have removed the claim from the Introduction and have instead incorporated the relevant findings (with appropriate statistical details) into the Results and Discussion sections, as reflected in the revised manuscript.

In the "Metric under model scaling" subsection of the Results, we have revised:

> *The proposed entity-centric metrics exhibit consistent positive alignment with model scaling across all five medical QA benchmarks (Table 3), indicating that EntQA better captures improvements in the quality of responses generated by larger models, which are generally associated with stronger reasoning ability, than conventional overlap-based metrics.*

We further revised the discussion of Table 2 as follows:

> *Overall, the two proposed EntQA metrics exhibit more consistently positive associations with accuracy across the five benchmarks, whereas the conventional baseline metrics often show weak, negative, or inconsistent correlations.*

Reviewer #2:

Comment R2C1: The Abstract should be reorganized. It is recommended to place greater emphasis on the key findings and results of the study.

Response R2C1: Thank you for the helpful suggestions. We have reorganized and substantially revised the abstract to place greater emphasis on the main findings and significance of the study rather than detailed methodological descriptions. In the revised version, we highlight the consistent positive associations between EntQA and model accuracy/scaling, its superior performance compared with conventional evaluation metrics, and its broader implications for assessing clinically reliable LLM-generated medical responses.

> *Large language models (LLMs) are increasingly evaluated for clinical question-answering (QA) tasks, yet benchmark accuracy alone provides limited assurance of clinical reliability. Existing evaluation metrics often fail to capture whether model reasoning preserves patient-specific context and diagnostic intent, while many require external references or manual annotation, limiting scalability and real-world applicability. To address this gap, this study proposes EntQA, a reference-free, entity-centric metric that evaluates how well LLM-generated responses retain clinically relevant biomedical concepts from patient backgrounds and diagnostic questions. Across five medical QA benchmarks and seven Qwen 2.5 Instruct models (0.5B--72B parameters), EntQA showed consistently positive associations with model accuracy and scaling, outperforming conventional overlap- and embedding-based metrics that frequently exhibited weak or negative correlations. Group-level correlations with accuracy reached Spearman r=0.9286, while correlations with model scale reached r=0.252. These findings suggest that EntQA provides a scalable and interpretable framework for assessing clinical fidelity and reasoning quality in healthcare LLMs without requiring gold-standard references or external evidence.*

Comment R2C2: The Introduction is too brief. Please provide a clearer description of the research motivation and objectives.

Response R2C2:We have substantially expanded the Introduction to provide a clearer description of the research motivation, the specific gaps in existing evaluation approaches, and the precise objectives of the present study.

> *Large language models (LLMs) have demonstrated strong performance on a range of medical question-answering (QA) tasks, including those that emulate licensing exams such as the USMLE. For instance, models like Med-PaLM have achieved expert-level accuracy by exceeding passing thresholds on USMLE-style questions, showcasing their potential to assist in diagnostic reasoning and knowledge retrieval. However, evaluating medical LLMs remains challenging because physicians don't judge medical advice solely by the correctness of the final diagnosis, but by whether the reasoning appropriately considers all clinically relevant patient information before arriving at a conclusion. In real-world clinical settings, overlooking seemingly minor details such as symptom duration, comorbidities or temporal progression, can substantially alter diagnostic interpretation and potentially mislead clinical decision-making. Therefore, evaluation metrics for medical QA should assess whether model-generated responses remain grounded in the clinically relevant evidence provided in the*

patient case, rather than relying solely on the factual correctness of isolated statements or superficial textual similarity.

Existing evaluation approaches often rely on coarse metrics such as accuracy or BLEU scores, which offer limited insight into whether model-generated responses faithfully adhere to patient-specific details. Consequently, these metrics may fail to identify cases where an LLM arrives at a seemingly correct conclusion while overlooking or fabricating critical clinical details from the patient case. This failure may allow clinically incomplete or insufficiently grounded reasoning to still receive favorable evaluation scores, potentially contributing to diagnostic errors, and limiting the trustworthiness of such systems in high-stakes medical settings.

Comment R2C3: Please incorporate more relevant literature from the past three years to strengthen the manuscript and support its contribution.

Response R2C3: We have added several recent references (2023–2025) on LLM hallucinations, reference-free evaluation, and entity-level grounding in medical QA to the Introduction

Prior work on evaluating LLMs in medical question answering initially focused on multiple-choice benchmarks such as MedQA [3] and USMLE-style tasks [4], where relying primarily on accuracy. Subsequent efforts expanded beyond exam-style setting to open-ended clinical tasks involving summarization, information extraction and diagnosis [5,6], as well as multilingual evaluation [7,8]. To assess those open-ended generation tasks, surface-level lexical metrics are adopted, such as BLEU [2], ROUGE [9]) and embedding-based metrics (e.g., BERTScore [10]). However, these approaches depends on expert-written reference answers, which are costly and difficult to obtain in clinical settings. Moreover, recent studies have questioned the reliability of these automatic metrics (e.g., ROUGE, BERTScore) since they disagree with clinician preferences and expert quality assessments in medical summarization tasks [11].

As a result, recent work shifted toward rubric-based evaluation frameworks that assess whether model outputs satisfy clinically meaningful criteria. Several studies introduced detailed evaluation rubrics covering dimensions such as factual accuracy, completeness, clarity, contextual awareness, instruction adherence, clinical reasoning, and patient safety [12,13]. These frameworks typically rely on clinician experts or advanced LLM judges to score responses against predefined standards. For example, prior work employed physician annotations to evaluate the quality, credibility, and safety of medical LLM responses [14,15], while other studies proposed structured human evaluation protocols for assessing hallucination, factual consistency,

diagnostic reasoning, and potential clinical harm [13,16].

Another line of work targets hallucination and reasoning validity by decomposing outputs into atomic facts or reasoning steps and verifying them against external knowledge bases [17–22].These approaches first extract fine-grained factual claims either through manual annotation [17,22] or automated LLM-based decomposition [18,19], and then assess whether each claim is supported by external evidence. Verification is typically performed by comparing claims against source documents, retrieved web evidence, or structured medical knowledge graphs [19–21]. More recently, reasoning-oriented benchmarks have extended evaluation beyond factual correctness to assess the quality of intermediate clinical reasoning steps themselves, including their completeness, efficiency, and consistency with established medical knowledge [19].

Comment R2C4: Please revise the manuscript by replacing "We" with a more formal academic expression such as "This study."

Response R2C4: The manuscript has been revised throughout to replace informal first-person expressions with more formal academic language and passive constructions where appropriate.

Comment R2C5: The Experimental settings section should be described more clearly to improve reproducibility and understanding.

Response R2C5: We have revised the Experimental Settings section to improve readability and reproducibility. Specifically, we now provide a more detailed description of the evaluation setup, including benchmark organization, response aggregation procedures, and statistical analyses.

To assess whether the proposed metrics are aligned with model correctness trends, two complementary analyses were conducted: (1) Group-level correlation.
For each benchmark, responses generated by the Qwen-2.5 Instruct models of increasing sizes were analyzed. For every model, the overall diagnostic accuracy and the average values of all evaluation metrics were computed, including the proposed entity-centric metrics for background coverage (EntQA_background or EntQA_b) and question coverage (EntQA_question or EntQA_q), as well as baseline metrics (BLEU, ROUGE, METEOR, and BERTScore). The monotonic association between model accuracy and average metric scores was examined using Spearman and Kendall correlation coefficients. This analysis evaluates whether models with higher diagnostic accuracy also tend to produce responses receiving consistently higher metric scores, thereby reflecting alignment between metric evaluations and overall model correctness trends.

(2) Case-level correlation.
To further evaluate the discriminative ability of the metrics at the individual response level,

responses generated by models of different sizes were aggregated within each benchmark. For each response, the binary diagnostic correctness label (correct vs. incorrect) and the corresponding metric scores were collected, and the association between correctness labels and metric values was quantified using point-biserial correlation. This analysis assesses whether correct responses systematically receive higher metric scores than incorrect ones, thereby evaluating the ability of the metrics to distinguish correct predictions from incorrect predictions.

Beyond correctness alignment, the extent to which the metrics reflect response quality improvements with increasing model capability was further investigated. Since model scale is commonly associated with stronger reasoning and generation ability, model size was used as a proxy for capability within the Qwen-2.5 Instruct family. Specifically, for each benchmark, the average metric scores were computed across all responses generated by each model. Spearman correlation was then calculated between model size and model-level average metric scores. This analysis examines whether the metrics consistently assign higher scores to responses generated by larger and more capable models, thereby evaluating whether metric behavior remains aligned with expected improvements in reasoning quality under model scaling.

Comment R2C6: The Results section is insufficiently detailed. It is recommended to expand the presentation and explanation of the study's outputs.

Response R2C6: We have expanded the case-study paragraph in the Results section for greater clarity. Specifically, we replaced the original example, which primarily served to illustrate the EntQA pipeline workflow, with a more diagnostically informative MedExpQA case that better demonstrates how EntQA reflects the quality and contextual grounding of LLM-generated responses. The revised analysis further illustrates how omission or misinterpretation of clinically informative entities may contribute to incorrect diagnostic reasoning and lower EntQA scores. To improve the focus and readability of the main manuscript, the original workflow-oriented figure (MedQA case) has been moved to the Appendix/Supplementary Materials.

A qualitative case study from MedExpQA (Figure 2) is presented to illustrate the practical utility of EntQA in identifying clinically relevant overlaps and gaps in LLM-generated responses. The scenario describes a 47-year-old man with myopia magna who underwent cataract surgery 2 years ago and presented to the emergency room with profound and painless loss of vision in the right eye. The diagnostic question pertains to the most probable diagnosis, with options: (A) Post-surgical endophthalmitis, (B) Retinal detachment, (C) Age-related macular degeneration, wet form, (D) Posterior vitreous detachment. The model's response suggests the correct answer 'retinal detachment', and the entity matching reveals the response retain most of the background information like 'high myopia', 'cataract surgery', '2 years', 'profound and painless loss', 'loss of vision', while only omitting 'emergency room'. In addition, the response covered one entity ("this symptomatology" as "this condition"). This yields coverage metrics of $EntQA\_b = 0.875$ (fraction of matched background entities in the answer) and $EntQA\_q = 0.5$ (fraction of matched question entities in the answer). By contrast, several lower EntQA responses for this question, whose scores are approximately 0.5-0.62), produced

incorrect predictions, frequently selecting post-surgical endophthalmitis (option A) instead of option B. Some cases ignored "2 years" timeline, implicitly assuming the surgery was recent, thus incorrectly favoring a postoperative diagnosis. Others failed to reason about "high myopia," a major risk factor for retinal detachment and a crucial clue supporting option B. These examples suggest that higher EntQA scores are generally associated with a more comprehensive use of clinically relevant contextual information and a higher probability of correct diagnostic reasoning.

Comment R2C7: In the Discussion section, please elaborate on the key findings, compare them with previous studies, and discuss the limitations of the research.

Response R2C7: We have added a new paragraph in the Discussion that directly compares EntQA with recent reference-free frameworks and have further elaborated on the limitations and future directions.

Compared with recent rubric-based evaluation frameworks and hallucination-oriented metrics, EntQA does not rely on expert-written references, proprietary LLM judges, or external medical knowledge bases. Instead, it performs entity-level matching and coverage analysis directly on the patient background, diagnostic question, and generated response, providing an interpretable assessment of clinical grounding and patient-specific reasoning.

Importantly, EntQA is intentionally designed to evaluate entity-level grounding and information coverage, focusing on whether clinically relevant details from patient backgrounds and diagnostic questions are adequately reflected in model-generated responses. This design is motivated by a common failure mode in medical LLMs, where models may generate plausible or fluent answers while overlooking critical patient-specific information, potentially leading to biased or incomplete clinical conclusions. In this context, low EntQA scores can help identify responses that fail to sufficiently incorporate key clinical evidence from the input. However, because EntQA focuses primarily on information retention and topical grounding, it does not fully capture higher-level aspects of response quality, such as logical coherence, causal reasoning, or factual correctness of medical interpretations. Consequently, responses preserving most of the contextual entities or with high entity coverage may still contain flawed reasoning or incorrect interpretations. EntQA should therefore be viewed as a complementary evaluation metric rather than a standalone assessment framework, and is most effective when combined with additional evaluation approaches such as reasoning-focused metrics, factuality assessment, structured error analysis, or expert clinician review.

Comment R2C8: Please include a Conclusion section to provide a clear summary of the study and its contributions.

Response R2C8: We have added a dedicated Conclusion section immediately before the Funding declaration.

This study introduces EntQA, a scalable, reference-free, entity-centric metric that evaluates clinical fidelity in LLM-generated medical responses by measuring retention of patient-specific and diagnostic entities. Across five established benchmarks and seven Qwen 2.5 Instruct models spanning three orders of magnitude in scale, EntQA demonstrated consistently positive and often statistically significant associations with both answer accuracy and model scaling, outperforming conventional lexical and embedding-based metrics. By delivering interpretable entity-level insights into omissions and contextual grounding, EntQA provides a practical tool for developers, researchers, and clinicians to assess and iteratively improve the trustworthiness of medical LLMs. The framework advances the development of clinically reliable AI systems and underscores the value of entity-centric evaluation in high-stakes healthcare domains.

Reviewer #3:

Comment R3C1: While the overall EntQA pipeline is clearly described, key implementation choices remain insufficiently specified. In particular, the biomedical NER model used for entity extraction is not described in enough detail, including its architecture, training data, or expected performance in this specific setting.

Response R3C1: We thank the reviewer for their constructive suggestions. We agree that providing specific details about the NER model is essential for the reproducibility and technical rigor of our work. In the revised manuscript, we have made changes to the **Methods** section in the main text (specifically under "Structured entity extraction") to detail the architecture, training data, or expected performance of our used NER model proposed by Raza et al.

The benchmark input was first separated into two components: the patient background (B) and the diagnostic question (Q). Clinically relevant entities were then extracted from the patient background, diagnostic question, and LLM-generated answer (A) using the Bio-Epidemiology-NER (BioEN) model proposed by Raza et al.. BioEN is a Transformer-based biomedical NER framework built on DistilBERT for sequence labeling of clinical entities. The model was trained on multiple annotated biomedical and epidemiological corpora, primarily the MACROBBAT 2020 dataset, which contains 200 manually annotated PubMed clinical case reports spanning diverse entity categories such as diseases, symptoms, diagnostic procedures, medications, demographic information, temporal expressions, and epidemiological concepts. Prior work reported macro- and micro-average F1 scores above 90% across several biomedical NER benchmarks, indicating strong generalization capability for clinical text extraction tasks. Because the benchmark used in this study consists of patient histories, diagnostic narratives, and LLM-generated clinical reasoning outputs, BioEN is well suited for extracting clinically relevant entities in this setting.

Comment R3C2: Similarly, the thresholds used for semantic matching (e.g., cosine similarity > 0.7 and lexical overlap > 30%) appear to be heuristically chosen, but no justification or sensitivity analysis is provided. It would strengthen the work to include empirical justification, ablation experiments, or robustness analyses demonstrating how sensitive EntQA is to these parameter choices.

Response R3C2: We agree that the choice of semantic matching thresholds should be further justified and evaluated for robustness. In the revised manuscript, we added a sensitivity analysis in the Appendix(Figure S1-S5) to examine the effects of different cosine similarity and lexical overlap thresholds on the EntQA metrics. The additional experiments show that although the absolute correlation values vary slightly across threshold settings, the overall trends and relative behaviors of the proposed entity-centric metrics remain largely consistent. In particular, EntQA continues to exhibit generally positive and more stable alignment with model correctness and model's reasoning ability (size as an indicator) compared with the baseline metrics across the tested parameter ranges. These results suggest that the proposed framework is reasonably robust to moderate variations in the heuristic matching thresholds, and that the main conclusions of the study do not depend on a single parameter choice.

Comment R3C3: In addition, while the manuscript acknowledges that EntQA focuses on entity coverage, the conceptual limitations of this design should be more explicitly emphasized. The metric primarily captures whether key entities from the input are retained in the generated response, but it does not assess higher-level aspects of response quality such as logical coherence, causal reasoning, or clinical correctness. As a result, a response could achieve a high EntQA score by mentioning many relevant entities while still being misleading or incorrect in its reasoning. This limitation is important, particularly in clinical contexts where accurate interpretation and reasoning are critical. The authors should more clearly position EntQA as a complementary metric rather than a standalone measure of response quality, and discuss how it might be combined with other evaluation approaches to provide a more comprehensive assessment.

Response R3C3: We agree that medical QA evaluation is inherently multi-dimensional, and that entity coverage alone cannot fully characterize overall response quality. Our intention with EntQA is not to replace reasoning- or correctness-oriented evaluation, but rather to address an important and often overlooked dimension of clinical LLM assessment: whether model-generated responses adequately incorporate clinically relevant information from the patient background and diagnostic question.

In response to the reviewer's suggestion, we made changes in the Discussion section (specifically after "EntQA has some limitations..") to more clearly position EntQA as a complementary evaluation metric focused on entity-level grounding and information coverage. We further clarified that a high EntQA score mainly indicates that a response covers more clinically relevant information from the input, making it more likely to be clinically appropriate, but it does not guarantee factual correctness or reasoning quality. Accordingly, EntQA is not intended to replace complementary evaluation approaches for factuality or clinical reasoning assessment.

> *Importantly, EntQA is intentionally designed to evaluate entity-level grounding and information coverage, focusing on whether clinically relevant details from patient backgrounds and diagnostic questions are adequately reflected in model-generated responses. This design is motivated by a common failure mode in medical LLMs, where models may generate plausible or fluent answers while overlooking critical patient-specific information, potentially leading to biased or incomplete clinical conclusions. In this context, low EntQA scores can help identify responses that fail to sufficiently incorporate key clinical evidence from the input. However, because EntQA focuses primarily on information retention and topical grounding, it does not fully capture higher-level aspects of response quality, such as logical coherence, causal reasoning, or factual correctness of medical interpretations. Consequently, responses preserving most of the contextual entities or with high entity coverage may still contain flawed reasoning or incorrect interpretations. EntQA should therefore be viewed as a complementary evaluation metric rather than a standalone assessment framework, and is most effective when combined with additional evaluation approaches such as reasoning-focused metrics, factuality assessment, structured error analysis, or expert clinician review.*
