# Supplementary material for: Entity-centric evaluation of large language model responses for medical question-answering tasks
Source: PLOS Digit Health. 2026 Oct 6;5(10):e0001752. doi: 10.1371/journal.pdig.0001752 (PMC13641718; doi:10.1371/journal.pdig.0001752)
Supplement: Supplementary file 2 [file pdig.0001752.s001.docx]

# S1 Appendix: supporting information

# **1 Sensitivity analysis of entity matching thresholds**

$EntQA$ determines entity matches using semantic similarity and lexical overlap constraints. The main experiments use an embedding similarity threshold of 0.7 and a token overlap threshold of 0.3. The robustness of $EntQA$ under different matching criteria was tested by varying the embedding similarity threshold from 0.60 to 0.90 and the token overlap threshold from 0.0 to 0.7. The resulting correlations under different parameter configurations are shown in Fig A-E.

Across benchmarks, varying threshold configurations primarily affects the magnitude of the correlations, while the overall qualitative conclusions presented in the main text remain stable. In particular, the proposed $EntQA$ metrics generally maintain stronger positive correlations with model accuracy and model scaling trends than conventional overlap-based metrics, which are predominantly negatively correlated across benchmarks. The following discussion uses MedQA, MedExpQA, and USMLE Step 2 as representative examples, while USMLE Step 1 and Step 3 exhibit similar trends.

Fig A presents the sensitivity analysis on MedQA. Conventional overlap-based metrics exhibited either weak or negative correlations with accuracy, with several metrics showing statistically significant negative correlations, including BLEU-1 $(-{0.8571}^{*})$ and ROUGE-1 $(-{0.8929}^{**}$). In contrast, the proposed $EntQA$ metrics maintained consistently positive correlations across threshold configurations. Given ${EntQA}_{b}$’s correlation with accuracy at the model level achieved ${0.857}^{*}$, it may further increase to ${0.929}^{*}.$

**Fig A: Sensitivity analysis of entity matching thresholds on MedQA.** Heatmaps show the correlations obtained under different embedding similarity thresholds (emb_threshold) and lexical overlap thresholds (tok_threshold) for ${EntQA}_{b}$ and ${EntQA}_{q}$. The analysis evaluates robustness across three correlation settings: model-level correlation with accuracy (Corr1), question-level point-biserial correlation (Corr2), and model size trend correlation (Corr3). Red boxes indicate the default threshold configuration used in the main experiments (emb_threshold=0.7, tok_threshold=0.3). Across threshold configurations, $EntQA$ maintains consistently positive correlations and preserves its relative advantages over conventional overlap-based metrics.

**Fig B: Sensitivity analysis of entity matching thresholds on MedExpQA.** Heatmaps show the correlations obtained under different embedding similarity thresholds (emb_threshold) and lexical overlap thresholds (tok_threshold) for ${EntQA}_{b}$ and ${EntQA}_{q}$. The analysis evaluates robustness across three correlation settings: model-level correlation with accuracy (Corr1), question-level point-biserial correlation (Corr2), and model size trend correlation (Corr3). Red boxes indicate the default threshold configuration used in the main experiments (emb_threshold=0.7, tok_threshold=0.3). Across threshold configurations, $EntQA$ maintains consistently positive correlations and preserves its relative advantages over conventional overlap-based metrics.

Fig B presents the sensitivity analysis on MedExpQA. Unlike conventional overlap-based metrics, which predominantly exhibited near-zero or negative correlations, $EntQA$ maintained consistently positive correlations across threshold configurations. Under the default setting (tok_threshold=0.3, emb_threshold=0.7), ${EntQA}_{b}$ achieved a model-level correlation of ${0.9286}^{**}$ with accuracy, and this value remained stable across the majority of threshold configurations. ${EntQA}_{q}$ achieved 0.536 under the default setting and varied between 0.536 and 0.750 across parameter settings. At the question level, ${EntQA}_{b}$ achieved ${0.093}^{**}$ under the default setting and increased to ${0.103}^{**}-{0.105}^{**}$ under several threshold configurations, exceeding the strongest baseline metric (BLEU-4: ${0.0987}^{**}$). ${EntQA}_{q}$ also became more positively correlated with correctness under alternative threshold settings, increasing from 0.022 to 0.034–0.037. For model scaling trends, ${EntQA}_{b}$ remained consistently positive and statistically significant across threshold configurations (${0.123}^{***}$–${0.198}^{***}$), while ${EntQA}_{q}$ generally remained above ${0.070}^{*}$ across parameter settings. Overall, the proposed entity-centric metrics consistently preserved stronger positive correlations with model accuracy and model scaling trends than conventional overlap-based metrics under varying threshold configurations.

**Fig C: Sensitivity analysis of entity matching thresholds on USMLE_STEP_1.** Heatmaps show the correlations obtained under different embedding similarity thresholds (emb_threshold) and lexical overlap thresholds (tok_threshold) for ${EntQA}_{b}$ and ${EntQA}_{q}$. The analysis evaluates robustness across three correlation settings: model-level correlation with accuracy (Corr1), question-level point-biserial correlation (Corr2), and model size trend correlation (Corr3). Red boxes indicate the default threshold configuration used in the main experiments (emb_threshold=0.7, tok_threshold=0.3). Across threshold configurations, $EntQA$ maintains consistently positive correlations and preserves its relative advantages over conventional overlap-based metrics.

**Fig D: Sensitivity analysis of entity matching thresholds on USMLE_STEP_2.** Heatmaps show the correlations obtained under different embedding similarity thresholds (emb_threshold) and lexical overlap thresholds (tok_threshold) for ${EntQA}_{b}$ and ${EntQA}_{q}$. The analysis evaluates robustness across three correlation settings: model-level correlation with accuracy (Corr1), question-level point-biserial correlation (Corr2), and model size trend correlation (Corr3). Red boxes indicate the default threshold configuration used in the main experiments (emb_threshold=0.7, tok_threshold=0.3). Across threshold configurations, $EntQA$ maintains consistently positive correlations and preserves its relative advantages over conventional overlap-based metrics.

Fig D presents the sensitivity analysis on USMLE_STEP_2. Across conventional overlap-based metrics, correlations with accuracy were predominantly negative, with several metrics exhibiting statistically significant negative correlations, including ROUGE-1, ROUGE-2, and BERTScore-F1 (all $-{0.8929}^{**}$). In contrast, $EntQA_{b}$ consistently preserved positive correlations across threshold configurations. In table 2 in the main text, $EntQA_{b}$ achieved a model-level correlation of 0.714, while alternative threshold settings increased the correlation to ${0.929}^{**}$. Across all tested parameter settings, the most of the correlation size remained within 0.643 – 0.929. $EntQA_{q}$ exhibited weaker correlations on this benchmark, varying between 0.143 and 0.429 at the model level and between −0.028 and 0.015 at the question level. Nevertheless, the magnitude of these negative associations remained substantially smaller than those observed for conventional overlap-based metrics. For model scaling trends, $EntQA_{b}$ remained consistently positive and statistically significant across threshold configurations (${0.113}^{**}$–${0.189}^{***}$), whereas conventional overlap-based metrics predominantly exhibited negative correlations. $EntQA_{q}$ achieved weaker but may have improved correlations under alternative threshold settings. Overall, although the correlations on USMLE_STEP_2 were weaker than those observed on the other benchmarks, the qualitative trends remained stable under varying threshold configurations, with $EntQA_{b}$ consistently preserving positive model-level and scaling-related correlations.

Fig E: Sensitivity analysis of entity matching thresholds on USMLE_STEP_3. Heatmaps show the correlations obtained under different embedding similarity thresholds (emb_threshold) and lexical overlap thresholds (tok_threshold) for ${EntQA}_{b}$ and ${EntQA}_{q}$. The analysis evaluates robustness across three correlation settings: model-level correlation with accuracy (Corr1), question-level point-biserial correlation (Corr2), and model size trend correlation (Corr3). Red boxes indicate the default threshold configuration used in the main experiments (emb_threshold=0.7, tok_threshold=0.3). Across threshold configurations, $EntQA$ maintains consistently positive correlations and preserves its relative advantages over conventional overlap-based metrics.

# **2 Illustration of EntQA pipeline**

A qualitative case study from MedQA (Fig F) is presented to illustrate the practical utility of EntQA in identifying clinically relevant overlaps and gaps in LLM-generated responses. The scenario describes a 15-year-old boy evaluated for learning disability, poor academic performance, and social interaction difficulties, with a history of hyperactivity, poor scores on reading and writing assessments, and molecular analysis showing increased CGG trinucleotide repeats. The diagnostic question pertains to the most likely physical examination finding in this patient, with options: (A) Frontal balding and cataracts, (B) Long face and large everted ears, (C) Almond-shaped eyes and downturned mouth, (D) Thin upper lip and receding chin. The model’s response suggests Fragile X Syndrome based on symptoms like intellectual disability, social interaction issues, and physical characteristics such as long face and large ears. However, entity matching reveals covered entities (e.g., “learning disability,” “hyperactivity,” “poor scores,” “molecular analysis,” “CGG trinucleotide repeats”) and uncovered entities (e.g., “physical examination,” “teachers,” “poor scores,” “assessments” from the background; “physical examination” from the question). This yields coverage metrics of ${EntQA}_{b}=0.62$ (fraction of matched background entities in the answer) and ${EntQA}_{q}=0.67$ (fraction of matched question entities in the answer), highlighting that the model’s summary misses key details like the long face and large everted ears, confirming option B as the correct answer.

**Fig F: Case study from MedQA.** This case demonstrates the entity-centric coverage metric’s ability to identify clinically relevant overlaps and gaps between the patient background, diagnostic question, and model-generated summary using biomedical named entity recognition (NER) and embedding-based entity matching.
