# Supplementary material for: Entity-centric evaluation of large language model responses for medical question-answering tasks
Source: PLOS Digit Health. 2026 Oct 6;5(10):e0001752. doi: 10.1371/journal.pdig.0001752 (PMC13641718; doi:10.1371/journal.pdig.0001752)
Supplement: Peer review history

# Peer review: Decision Letter 0

**DOI:** 10.1371/journal.pdig.0001752.r001

**Authors:** Dhiya Al-Jumeily OBE

6 Apr 2026

Response to Reviewers Revised Manuscript with Track Changes Manuscript **Journal Requirements:**

1. Please amend your detailed Financial Disclosure statement. This is published with the article. It must therefore be completed in full sentences and contain the exact wording you wish to be published.

i. Please clarify all sources of financial support for your study. List the grants, grant numbers, and organizations that funded your study, including funding received from your institution. Please note that suppliers of material support, including research materials, should be recognized in the Acknowledgements section rather than in the Financial Disclosure.

ii. State the initials, alongside each funding source, of each author to receive each grant. For example: "This work was supported by the National Institutes of Health (####### to AM; ###### to CJ) and the National Science Foundation (###### to AM)."

iii. State what role the funders took in the study. If the funders had no role in your study, please state: “The funders had no role in study design, data collection and analysis, decision to publish, or preparation of the manuscript.”

iv. If any authors received a salary from any of your funders, please state which authors and which funders.

2. Please send a completed 'Competing Interests' statement, including any COIs declared by your co-authors. If you have no competing interests to declare, please state "The authors have declared that no competing interests exist". Otherwise please declare all competing interests beginning with the statement "I have read the journal's policy and the authors of this manuscript have the following competing interests:"

3. Please note that your Data Availability Statement is currently missing the repository name and/or the DOI/accession number of each dataset OR a direct link to access each database. If your manuscript is accepted for publication, you will be asked to provide these details on a very short timeline. We therefore suggest that you provide this information now, though we will not hold up the peer review process if you are unable.

4. We ask that a manuscript source file is provided at Revision. Please upload your manuscript file as a .doc, .docx, .rtf or .tex.

5. Please upload separate figure files in .tif or .eps format. Also, remove the figures from your manuscript file but keep the legends.

For more information about figure files please see our guidelines:

https://journals.plos.org/digitalhealth/s/figures

https://journals.plos.org/digitalhealth/s/figures#loc-file-requirements

6. Please provide an Author Summary. This should appear in your manuscript between the Abstract (if applicable) and the Introduction, and should be 150–200 words long. The aim should be to make your findings accessible to a wide audience that includes both scientists and non-scientists. Sample summaries can be found on our website under Submission Guidelines:

https://journals.plos.org/digitalhealth/s/submission-guidelines#loc-parts-of-a-submission

7. Some material included in your submission may be copyrighted. According to PLOS’s copyright policy, authors who use figures or other material (e.g., graphics, clipart, maps) from another author or copyright holder must demonstrate or obtain permission to publish this material under the Creative Commons Attribution 4.0 International (CC BY 4.0) License used by PLOS journals. Please closely review the details of PLOS’s copyright requirements here: PLOS Licenses and Copyright. If you need to request permissions from a copyright holder, you may use PLOS's Copyright Content Permission form.

Please respond directly to this email or email the journal office and provide any known details concerning your material's license terms and permissions required for reuse, even if you have not yet obtained copyright permissions or are unsure of your material's copyright compatibility.

Potential Copyright Issues:

a. Figures 1, 2: Please confirm whether you drew the images / clip-art within the figure panels by hand. If you did not draw the images, please provide (a) a link to the source of the images or icons and their license / terms of use; or (b) written permission from the copyright holder to publish the images or icons under our CC-BY 4.0 license. Alternatively, you may replace the images with open source alternatives. See these open source resources you may use to replace images / clip-art:

- https://commons.wikimedia.org

- https://openclipart.org/

**Additional Editor Comments (if provided):** **Reviewers' Comments:**

**Comments to the Author**

1. Does this manuscript meet PLOS Digital Health’s publication criteria?

Reviewer #1: Yes

Reviewer #2: Yes

Reviewer #3: Yes

**********

2. Has the statistical analysis been performed appropriately and rigorously?

Reviewer #1: Yes

Reviewer #2: I don't know

Reviewer #3: Yes

**********

3. Have the authors made all data underlying the findings in their manuscript fully available (please refer to the Data Availability Statement at the start of the manuscript PDF file)?

The PLOS Data policy

Reviewer #1: Yes

Reviewer #2: Yes

Reviewer #3: Yes

**********

4. Is the manuscript presented in an intelligible fashion and written in standard English?

Reviewer #1: Yes

Reviewer #2: No

Reviewer #3: Yes

**********

**Reviewer #1:**  First of all, thank you for giving me the opportunity to review your article.

I must admit that I genuinely appreciate the method you propose for performance measurement from a logical standpoint. We already know that n-gram techniques remain superficial. Unfortunately, BERTScore’s single evaluation method along the hypothesis-reference axis cannot go beyond general similarity. The bidirectional evaluation in the embedding model you propose represents an innovative and valuable approach for factual grounding and relevance.

However, I believe it would be beneficial for both readers and researchers who wish to replicate the test if you could clarify some important points:

1. In the manuscript, you state that for the system to perform semantic matching, “the cosine similarity must be greater than 0.7 and the word overlap must be over 30%.” However, I did not see an analysis showing how changing these values affects EntQA’s performance. I noted that you mentioned these thresholds might require dataset-specific tuning to optimize performance, but I am left wondering whether these parameters manipulate the results and, in datasets where there are no definitive truths, how these adjustments should be made.

2. In the article, the r value for EntQAb in the USMLE Step 3 is reported as 0.0490, but Table 2 lists it as 0.09. Which one is correct?

3. Do you have any explanation for why BLEU-4 shows a higher correlation in the case-level accuracy test?

4. Although you have noted that the results are weak and inconsistent, even the highest EntQA correlation in your point-biserial correlation table remains below 0.1. Yes, the p-value appears statistically significant, but I believe this may be related to the sample size. Therefore, I think it would be more accurate to evaluate the results from this perspective.

5. Additionally, an assessment is needed regarding the lack of significant correlation in USMLE Step 1 and 2 in Table 2. I couldn’t make sense of the difference between them and Step 3.

6. Additionally, in the article, you state that “EntQAb still maintains a positive trend (0.7143 / 0.619),” but it is clear from Table 1 that no statistical significance is found.

7. In the Introduction section, on lines 41–42, you have used the phrase “where it consistently showed higher correlation with model accuracy and reasoning ability.” It would be more appropriate to include this in the Results and/or Discussion section.

**Reviewer #2:**  1.The Abstract should be reorganized. It is recommended to place greater emphasis on the key findings and results of the study.

2.The Introduction is too brief. Please provide a clearer description of the research motivation and objectives.

3.Please incorporate more relevant literature from the past three years to strengthen the manuscript and support its contribution.

4.Please revise the manuscript by replacing “We” with a more formal academic expression such as “This study.”

5.The Experimental settings section should be described more clearly to improve reproducibility and understanding.

6.The Results section is insufficiently detailed. It is recommended to expand the presentation and explanation of the study’s outputs.

7.In the Discussion section, please elaborate on the key findings, compare them with previous studies, and discuss the limitations of the research.

8.Please include a Conclusion section to provide a clear summary of the study and its contributions.

**Reviewer #3:**  The manuscript would benefit from additional detail to improve methodological clarity and reproducibility. While the overall EntQA pipeline is clearly described, key implementation choices remain insufficiently specified. In particular, the biomedical NER model used for entity extraction is not described in enough detail, including its architecture, training data, or expected performance in this specific setting. Given that the proposed metric directly depends on the quality of extracted entities, any errors or biases in the NER step could substantially affect the resulting coverage scores and downstream conclusions. Similarly, the thresholds used for semantic matching (e.g., cosine similarity > 0.7 and lexical overlap > 30%) appear to be heuristically chosen, but no justification or sensitivity analysis is provided. It would strengthen the work to include empirical justification, ablation experiments, or robustness analyses demonstrating how sensitive EntQA is to these parameter choices.

In addition, while the manuscript acknowledges that EntQA focuses on entity coverage, the conceptual limitations of this design should be more explicitly emphasized. The metric primarily captures whether key entities from the input are retained in the generated response, but it does not assess higher-level aspects of response quality such as logical coherence, causal reasoning, or clinical correctness. As a result, a response could achieve a high EntQA score by mentioning many relevant entities while still being misleading or incorrect in its reasoning. This limitation is important, particularly in clinical contexts where accurate interpretation and reasoning are critical. The authors should more clearly position EntQA as a complementary metric rather than a standalone measure of response quality, and discuss how it might be combined with other evaluation approaches to provide a more comprehensive assessment.

**********

what does this mean?). If published, this will include your full peer review and any attached files.

**Do you want your identity to be public for this peer review?**  If you choose “no”, your identity will remain anonymous but your review may still be made public.

For information about this choice, including consent withdrawal, please see our Privacy Policy

Reviewer #1: No

Reviewer #2: No

Reviewer #3: No

**********

**Figure resubmission:**

**Reproducibility:**

To enhance the reproducibility of your results, we recommend that authors of applicable studies deposit laboratory protocols in protocols.io, where a protocol can be assigned its own identifier (DOI) such that it can be cited independently in the future. Additionally, PLOS ONE offers an option to publish peer-reviewed clinical study protocols. Read more information on sharing protocols at https://plos.org/protocols?utm_medium=editorial-email&utm_source=authorletters&utm_campaign=protocols

# Author comment: Author response to Decision Letter 1

**DOI:** 10.1371/journal.pdig.0001752.r002

14 Jun 2026

Attachment Submitted filename: PLOS_health_response_letter.pdf

# Peer review: Decision Letter 1

**DOI:** 10.1371/journal.pdig.0001752.r003

**Authors:** Dhiya Al-Jumeily OBE

8 Sep 2026

Entity-centric evaluation of large language model responses for medical question-answering tasks

PDIG-D-25-01161R1

Dear Dr. Kolachalama,

We are pleased to inform you that your manuscript 'Entity-centric evaluation of large language model responses for medical question-answering tasks' has been provisionally accepted for publication in PLOS Digital Health.

Before your manuscript can be formally accepted you will need to complete some formatting changes, which you will receive in a follow-up email from a member of our team.

Please note that your manuscript will not be scheduled for publication until you have made the required changes, so a swift response is appreciated.

IMPORTANT: The editorial review process is now complete. PLOS will only permit corrections to spelling, formatting or significant scientific errors from this point onwards. Requests for major changes, or any which affect the scientific understanding of your work, will cause delays to the publication date of your manuscript.

If your institution or institutions have a press office, please notify them about your upcoming paper to help maximize its impact. If they'll be preparing press materials, please inform our press team as soon as possible -- no later than 48 hours after receiving the formal acceptance. Your manuscript will remain under strict press embargo until 2 pm Eastern Time on the date of publication. For more information, please contact digitalhealth@plos.org.

Thank you again for supporting Open Access publishing; we are looking forward to publishing your work in PLOS Digital Health.

Best regards,

Dhiya Al-Jumeily OBE, PhD

Section Editor

PLOS Digital Health

***********************************************************

**Additional Editor Comments (if provided):**

**Reviewer Comments (if any, and for reference):**

Reviewer's Responses to Questions

**Comments to the Author**

Reviewer #2: All comments have been addressed

Reviewer #3: All comments have been addressed

**********

publication criteria?

Reviewer #2: (No Response)

Reviewer #3: Yes

**********

3. Has the statistical analysis been performed appropriately and rigorously?

Reviewer #2: (No Response)

Reviewer #3: Yes

**********

4. Have the authors made all data underlying the findings in their manuscript fully available (please refer to the Data Availability Statement at the start of the manuscript PDF file)?

The PLOS Data policy

Reviewer #2: (No Response)

Reviewer #3: Yes

**********

5. Is the manuscript presented in an intelligible fashion and written in standard English?

Reviewer #2: (No Response)

Reviewer #3: Yes

**********

Reviewer #2: (No Response)

Reviewer #3: Overall, I think the authors have responded very thoroughly to the reviewers. Most reviewer concerns are fully addressed, and I would likely recommend acceptance.

**********

what does this mean?). If published, this will include your full peer review and any attached files.

**Do you want your identity to be public for this peer review?**  If you choose “no”, your identity will remain anonymous but your review may still be made public.

For information about this choice, including consent withdrawal, please see our Privacy Policy

Reviewer #2: None

Reviewer #3: No

**********
